# Following Ariadne's thread: a new perspective on RBR ubiquitin ligases

**DOI:** 10.1186/1741-7007-10-24

**Published:** 2012-03-15

**Authors:** Dawn M Wenzel, Rachel E Klevit

**Affiliations:** 1 Department of Biochemistry, University of Utah School of Medicine, 15 N. Medical Drive East, Salt Lake City, UT 84112-5650, USA; 2 Department of Biochemistry, University of Washington, Seattle, Washington 98195, USA

## Abstract

Ubiquitin signaling pathways rely on E3 ligases for effecting the final transfer of ubiquitin from E2 ubiquitin conjugating enzymes to a protein target. Here we re-evaluate the hybrid RING/HECT mechanism used by the E3 family RING-between-RINGs (RBRs) to transfer ubiquitin to substrates. We place RBRs into the context of current knowledge of HECT and RING E3s. Although not as abundant as the other types of E3s (there are only slightly more than a dozen RBR E3s in the human genome), RBRs are conserved in all eukaryotes and play important roles in biology. Re-evaluation of RBR ligases as RING/HECT E3s provokes new questions and challenges the field.

## E3 ligases: historically a two-party system

**Figure 1:**
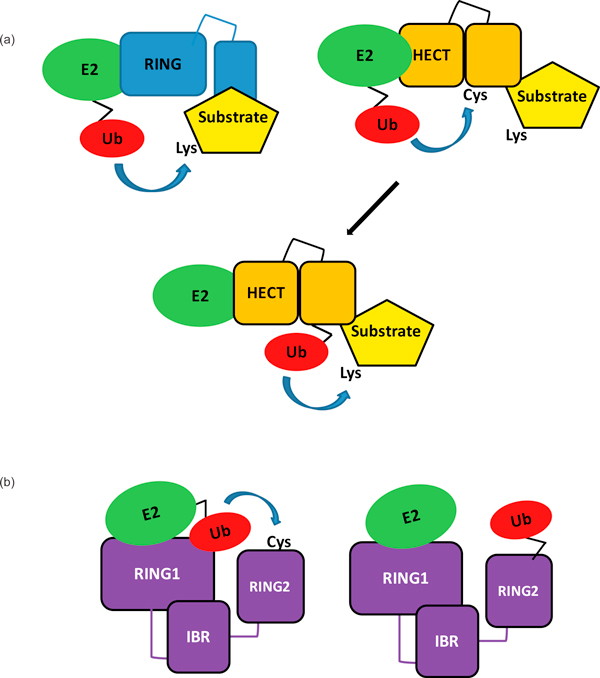
**RING and HECT-type mechanisms of ubiquitin transfer**. **(a) **On the left, a RING E3 ligase (blue) is shown bound to a ubiquitin-conjugated E2, from which the ubiquitin is transferred to a lysine on the substrate. On the right, a HECT E3 ligase (orange) is shown bound to a ubiquitin-conjugated E2, from which ubiquitin is first transferred to the active-site cysteine of the E3, and is then transferred to a lysine on the substrate bound to the E3 (lower panel). **(b) **Proposed mechanism for RBR ubiquitin transfer. RBR ligases combine features of both RING- and HECT-type ligases. The ubiquitin-conjugated E2 binds to the RING1 domain of the RBR E3 ligase. The ubiquitin is then transferred from the E2 to the E3 RING2 domain from which it is transferred to the substrate.

Ubiquitination is the process by which proteins are selectively targeted for a variety of cellular fates. This post-translational modification is carried out by a trio of enzymes: an E1 ubiquitin-activating enzyme, an E2 ubiquitin-conjugating enzyme, and an E3 ubiquitin ligase. In most cases, E3 ubiquitin ligases assume the role of transferring activated ubiquitin from a restricted cohort of E2s to specific substrates. In a given genome, putative E3s greatly outnumber E2s, underscoring their role in substrate selection. For example, in humans, there are over 600 E3 ubiquitin ligases and fewer than 40 E2s [1]. On the basis of their mechanism and structure, E3 ligases have historically been classified into two families, the HECT- and RING/UBOX-type ligases (Figure 1). Recently we determined that Ariadne, the defining member of a subclass of RING-containing E3 ligases known as RING-between-RINGs (RBRs), blurs the line between RING and HECT-type E3s.

Eukaryotic E3 ubiquitin ligases are generally identified by the presence of either a HECT or a RING domain. The features of each type of domain are well defined and are readily predictable by primary sequence analysis. RINGs are characterized by a regular spacing of conserved cysteines and histidines which bind two Zn^2+ ^ions that stabilize the overall structure of this domain, allowing for recognition and activation of E2 Ub-conjugating enzymes [2]. HECT domains are identified on the basis of their similarity to the founding member of the family, E6AP. In contrast to RING domains, which can occur at any position within a given protein, all known HECT domains are found at the carboxy-terminal end of their respective proteins. The HECT domain has a bilobal structure: the lobe at the amino-terminal end of the domain (the N-lobe) serves as the E2-binding domain, and the lobe at the carboxyl terminus (the C-lobe) contains the catalytic cysteine.

There are two general mechanisms by which the ultimate substrate-ubiquitin isopeptide adduct is formed. An essential difference between the two mechanisms is the location of activated ubiquitin at the final transfer step (Figure 1a). In reactions involving RING/UBOX-type ligases, the ubiquitin is attached to an E2 to form an E2~Ub thioester conjugate, and the E3 binds both substrate and the E2~Ub simultaneously to promote the aminolysis reaction in which ubiquitin is transferred to a lysine on a substrate. By an as yet undetermined mechanism, E3 binding enhances the reactivity of the E2~Ub thioester bond to allow for aminolysis [3,4]. Catalytic residues have not been identified for RING/UBOX type ligases and are presumed not to exist. In reactions involving HECT E3s, ubiquitin is transferred from an E2~Ub to form an E3~Ub thioester conjugate and the final transfer step occurs directly from the E3 active site to a substrate lysine. Thus, the two mechanisms differ in terms of the identity of the active site that is responsible for the aminolysis: it is the E2 active site in RING/UBOX-catalyzed reactions and it is the E3 active site in HECT-catalyzed reactions. Substrates may be mono-ubiquitinated at one or more sites; or poly-ubiquitin chains may be attached to them. Poly-ubiquitin chains may be of eight known topologies determined by their distinct linkages, and named K48, K63, K11, K27, K29, K33, and K6 chains according to the lysine residue through which the ubiquitins are linked to one another; or linear chains when the linkage is between the carboxyl terminus of one ubiquitin and the amino terminus of the next

Because the E2 active site is responsible for aminolysis in RING/UBOX-catalyzed reactions, it follows that the product produced by RING/UBOX-type ligases, be it mono-ubiquitination or a poly-ubiquitin chain of a specific topology, is determined in large part by the identity of the E2 involved in the reaction. A RING-type E3 that can bind a diverse set of E2s has the potential to produce several distinct types of ubiquitination products. This phenomenon has been demonstrated for a growing number of RING-type ligases such as BRCA1/BARD1 and the APC, which can produce poly-ubiquitin of K63 and K48 linkages, as well as mono-ubiquitin, depending on the E2 [5,6]. In contrast, it is the identity of the HECT-type E3 itself that determines the type of ubiquitin modifications conferred on substrates by this class of E3 ligases, and residues in the active-site-containing C-lobe have been shown to determine the type of ubiquitination signal generated by HECT-type E3s [7].

## RBR E3s: Reaching across the aisle

RBR E3s were originally identified by virtue of their RING domains, but the presence of two additional domains, IBR and RING2, define them as a subclass [8,9]. RBR-type ligases are defined by a trio of closely spaced domains: 1) a canonical amino-terminal RING domain, dubbed RING1, 2) an in-between RING domain (IBR), and 3) a domain named RING2 [10]. As in the case of canonical RING domains, RBR domains are not limited to any particular location within the proteins that contain them. Although RING1 domain sequences follow the cysteine and histidine patterns typical of RINGs from other E3 ligase families, the IBR and RING2 domain do not, and these two domains are unique to RBR-type proteins. RBR E3 ligases are found throughout eukaryotes with two members in yeast and thirteen in human [10].

Despite their strong persistence throughout evolution, most RBR E3s are not well understood, and their substrates and E2 partners are poorly defined. Members of this E3 family mediate diverse processes that include the regulation of translation and the activation of NF-κB signaling, among others [11,12]. The most studied RBR E3 is Parkin, because of its association with Parkinson's disease [13]. The list of Parkin substrates continues to grow, although it is not clear which are critical for understanding the death of dopaminergic neurons, the hallmark of Parkinson's disease. The first substrates reported for Parkin were α-synuclein, Pael-R, and CDCrel-1, all of which accumulate in patients with heritable Parkinson's disease [14-16]. These substrates suggest a role for Parkin in the clearance of misfolded or aggregation-prone proteins. Additionally, Parkin is recruited to mitochondria where it is thought to regulate turnover of damaged mitochondria. Proteins that regulate mitochondrial morphology, mitofusin-1 and mitofusin-2, are substrates of Parkin ubiquitination [17,18].

Several RBR ligases have roles in regulating immune signaling. Overexpression of RNF216 (TRIAD3, ZIN) enhances the degradation of specific Toll-like receptors, with co-ordinate down-regulation of Toll-like receptor signaling [19]. RNF216 may function to attenuate the host response to viral invasion, as RNF216 ubiquitinates TRAF3, a potentiator of the anti-viral response, and targets it for proteasomal degradation [20]. The RBR proteins Rbck1 (HOIL-1) and RNF31 (HOIP) form a heterodimeric E3 ligase that function together with the protein SHARPIN to generate linear ubiquitin chains [21,22]. Linearly-linked poly-ubiquitin chains specifically recruit components of the NF-κB signaling pathway including NEMO to promote the phosphorylation and subsequent degradation of inhibitors of NF-κB.

Yeast contain two RBR proteins, orthologs for Ariadne (ARIH1) and RNF14 (ARA54). It has been suggested that the extreme conservation of RBR genes from the earliest eukaryotes to human may indicate that these proteins assume a housekeeping role [23]. Human RNF14 was first identified through a yeast two hybrid screen for proteins that bind to the androgen receptor, and is thought to be a co-activator of androgen receptor function [24]. The yeast ortholog to RNF14 may have a function in the regulation of translation termination [25]. The *Drosophila *protein Ariadne, which was the first member of the RBR family to be described, is important for the development of the fly nervous system: neuronal differentiation is disrupted in Ariadne mutants because of a failure in axon guidance [9]. Ariadne was named after the Greek goddess Ariadne, whose thread was said to help guide Theseus out of the Minotaur's labyrinth.

Ariadne and other RBR-type E3s have been presumed to be RING-like in both structure and mechanism because of the similarities between RING1 and canonical RING domains. Unexpectedly, we discovered that the human RBR E3 HHARI (human homolog of Ariadne) functions in a way that is analogous to that of a HECT-type ligase, in that it forms an obligate thioester bond with ubiquitin before it is transferred to the substrate [3] (Figure 1b). A cysteine in the RING2 domain that is highly conserved among all RBR-type E3s functions like a HECT active site cysteine: when this cysteine is mutated to serine, an oxy-ester-linked HHARI~Ub intermediate can be trapped. Mutation of the analogous cysteine residue (C431) in RING2 of Parkin disrupts ubiquitin transfer mediated by this RBR E3, suggesting that this mode of ubiquitin transfer is a general feature of RBR E3s. Because they contain both a RING-type E2-binding domain at their amino-terminal end and an active-site-containing domain at their carboxy-terminal end, we proposed that RBRs can be thought of as RING-HECT hybrids.

## Domain and structural analysis of RBRs

To date, structural insights into the RBR ligase supradomain are limited to structures solved for individual component domains: there are two structures of IBR domains, and one structure each of a RING1 and RING2 domain [26,27] [PDB ID 1WIM, PDB ID 2CT7]. The dearth of structural information is probably due to the difficulty of expressing and purifying these proteins in large amounts. With such a small sampling, it is unclear whether these structures will be representative of the entire family of RBR ligases. However, the sequence similarity of these domains among human members of the RBR ligase family suggests we may expect to see similar structures.

**Figure 2:**
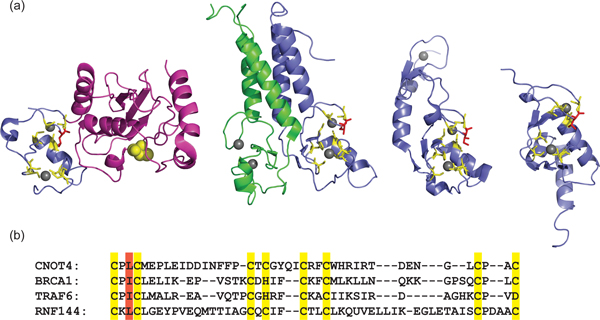
**RING1 of RBRs maintains features characteristic of canonical RINGs**. **(a) **Structures of RING domains are displayed with Zn^2+ ^coordinating residues as yellow sticks and Zn^2+ ^ions displayed as grey spheres. A conserved hydrophobic residue important for E2 binding is shown as orange sticks. The structures are (from left to right) the E3 ligase CNOT4 (blue) bound to the E2 UbcH5b (purple) (PDB ID 1UR6) (the E2 active site is shown as yellow spheres); the heterodimeric RING E3 ligase BRCA1 (blue)/BARD1 (green) (PDB ID 1JM7); TRAF6 (PDB ID 3HCT); RING1 of the RBR E3 RNF144 (PDB ID 1WIM). **(b) **Multiple sequence alignment of the RING domains of CNOT4, BRCA1, TRAF6, and RNF144. Coloring in the multiple sequence alignment corresponds with the colors in the structures, highlighting residues important for Zn^2+ ^coordination and E2 binding. Sequences were aligned using CLUSTALW and manually adjusted based on structure [44].

Though limited, the available structures do provide some functional insights. The structure of the RNF144 RING1 is similar to that of other RING-type E3s such as BRCA1, with the E2-binding interface readily apparent (PDB ID 1WIM, Figure 2). E2-E3 disrupting mutations that are structurally analogous to those in *bona fide *RING-type E3s abrogate the binding of the E2 UbcH7 to HHARI [5,28,29]. Although the E2-E3 binding interactions of RBR E3 ligases seem to be analogous to those of the RING E3 ligases, the functional consequence of the interaction is not. Whereas binding of the BRCA1/BARD1 RING domain leads to a significant enhancement of the thioester reactivity of the E2~Ub conjugate (in this case UbcH5c~Ub) towards lysine, no enhancement of E2 reactivity was detected for a construct composed of the RING1 and IBR domains of HHARI [3]. Thus, although the RING1 domain of HHARI serves as an E2-binding domain, it lacks the catalytic capacity of other canonical RING domains. Whether this is a general feature of the RING1 domains of RBRs remains to be tested experimentally.

**Figure 3:**
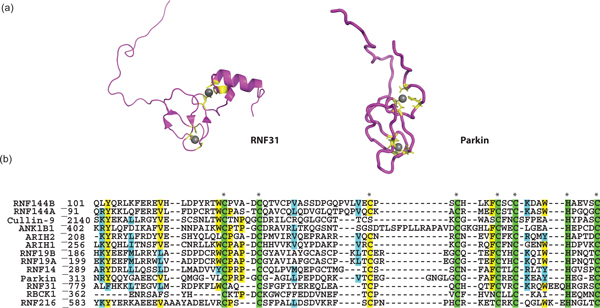
**Conservation of the IBR domain**. **(a) **Structures of IBR domains solved to date from RNF31 (left) (PDB ID 2CT7) and Parkin (right) (PDB ID 2JMO). **(b) **Multiple sequence alignment of the IBR domain from human RBR ligases. Residue numbers are shown at the beginning of the alignment. Sequences were aligned using CLUSTALW [44]. Swiss-Prot numbers for sequences used in multiple sequence alignments are as follows: Cullin-9: Q81WT3, Parkin: O60260, ANKIB1: Q9P2G1, ARIH1: Q9Y4X5, ARIH2: O95376, RBCK1: Q9BYM8, RNF144A: P50876, RNF144B:Q7Z419, RNF19A:Q9NV58, RNF19B: Q6ZMZ0, RNF216: Q9NWF9, RNF14: Q9UBS8, and RNF31: Q96EP0.

The function of the IBR domains of RBR ligases is even less clear. The IBR domains of Parkin and RNF31 (HOIP) are structurally sparse outside of the two Zn^2+ ^binding-centers (Figure 3) [26]. Shaw and colleagues noted that the amino- and carboxy-termini of the IBR domain are close to one another and may therefore serve to bring the RING1 and RING2 domains together [26]. Such a function would be analogous to that of the flexible linker found between the amino-lobe and the carboxy-lobe in HECT-type E3s, which allows conformational changes essential for E3 activity. If so, one would predict that mutations that either reduce the flexibility of the putative linker region or change the orientation of the two RING domains would disrupt ubiquitin transfer, as reported for the HECT ligase WWP1 [30]. However, the structural conservation of the IBRs in terms of Zn^2+^-binding residues, number of amino acids, and the fact that this domain is found almost exclusively in RBR E3s, suggest that the domain is unlikely to serve merely as an elaborate linker.

**Figure 4:**
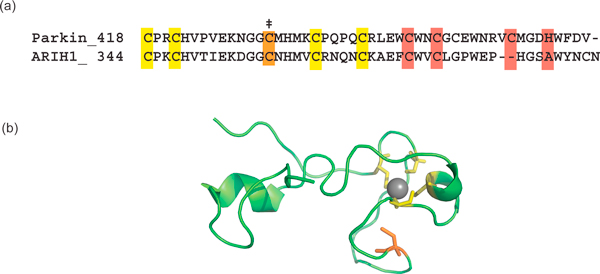
**Comparison of HHARI RING2 with Parkin RING2**. **(a) **Multiple sequence alignment of HHARI RING2 and Parkin RING2. Zn^2+^-liganding residues determined structurally for HHARI RING2 are denoted in yellow. Potential additional Zn^2+ ^coordinating residues in Parkin as proposed by Rankin *et al. *[34] are highlighted in red. The active site cysteine is denoted by a double dagger. **(b) **The structure of HHARI RING2 displays Zn^2+^-liganding residues as yellow sticks. The active site cysteine is shown as orange sticks (PDB ID 1WD2). Sequences were aligned using CLUSTALW [44].

Our finding that Parkin RING2 residue C431 is an active site cysteine clarifies several observations about Parkin activity, while raising new questions about the structure and mechanisms of this family of ligases. The Parkin mutation C431F gives rise to a loss-of-function phenotype and is associated with juvenile onset Parkinson's disease, a phenotype that was earlier attributed to structural destabilization, under the presumption that C431 was involved in Zn^2+ ^binding [31]. More recent experiments, however, in which the conserved cysteines of Parkin were systematically mutated to alanine, showed that most mutants were insoluble and mislocalized, except the C431A mutant, suggesting that this residue is not part of the Zn^2+ ^coordination network of Parkin [32]. The only RING2 structure to date is of HHARI RING2 [27] (Figure 4). Unlike the RING1 domain, whose structure led to the notion that the mechanism of the RBR ligases would be like that of the canonical RING ligases, the HHARI RING2 domain structure looks nothing like a canonical E2-binding RING: we discuss below the implications of this structural dissimilarity. Despite the presence of six well conserved cysteines, the structure binds only one Zn^2+ ^ion, leaving two cysteine residues unliganded [27]. We have identified one of the free cysteines, C357, as the active site cysteine of HHARI. The single Zn^2+^-binding site in HHARI RING2 contrasts with electrospray mass spectrometry data that predict two Zn^2+ ^ions for Parkin RING2 [33]. The coordination of Zn^2+ ^residues in Parkin may well differ from that of HHARI, and how this is accomplished remains to be determined structurally. Rankin and colleagues propose a Parkin RING2 model with two Zn^2+^-binding sites, composed of residues C418, C421, C441, C436 and C446, C449, H461, C457 [34]. This model leaves C431 free to form a thioester bond with ubiquitin (Figure 4).

## RBR isopeptide catalysis

The observation that RBR ligases function through a HECT-like mechanism implies that there are additional residues near the active site cysteine that facilitate isopeptide catalysis with a substrate lysine. However, if studies with HECT E3s are any indication, these may be difficult to identify. Aside from the active site cysteine, few catalytic residues have been identified within HECT domain ligases. Studies involving a bacterial HECT-like E3, SopA, identified a conserved motif among HECT domain active sites surrounding the active site cysteine, LXXShTCfXn (where upper case letters indicate invariant residues, lower case letters indicate conserved residues and X indicates a variable position) [35]. Mutation of the conserved leucine or threonine to an alanine decreases activity [35]. Removal of a highly conserved phenylalanine in the carboxy-terminal tail of HECT domain ligases abolishes the ability of HECT E3s to modify lysines on substrates, but does not interfere with transthiolation from E2 to the HECT active site cysteine [36]. Whether the conserved phenylalanine in HECT domains plays a direct role in catalysis remains to be seen. It is not clear if RBR-type E3s will use a similar mechanism for function. The lack of similarity between HECT E3 C-lobes and RBR RING2s at the sequence level suggests it will be challenging to identify residues that are important for isopeptide catalysis on the basis of our current knowledge. Residues around the RBR active site cysteine do not follow the LXXShTCfXn motif, and as the role of the carboxy-terminal phenylalanine in HECT-type ligases is not well understood, it is impossible to know if RBR-type ligases have residues that fulfill a similar function. The possibility remains that residues in other domains of the RBR may contribute to the activity of RING2. Structural and biochemical characterization of RING2 in context of the RING1 and IBR domains may be key to future progress in this respect.

Like HECT ligases, the ubiquitination products generated by RBRs are independent of E2 identity and are likely to be an intrinsic property of each RBR E3. To date, polyubiquitin chains with linear, Lys48, and Lys63 linkages, multiple mono-ubiquitination, and mono-ubiquitination have all been reported as products of RBR ligases [37-41]. Although Parkin itself has been reported to form many types of products, a recent study suggests that Parkin products can be influenced by the fusion of artificial molecular tags, or truncation of constructs, so previous results should be viewed in this light [42]. The RBR proteins RBCK1 (HOIL-1) and RNF31 (HOIP) form a complex known as LUBAC (Linear Ubiquitin Assembly Complex) and produce linear polyubiquitin chains with the human E2s UbcH7, UbcH5, and Ube2k [37]. To date, no HECT-type or canonical RING-type E3s have been reported to build linear poly-ubiquitin chains, suggesting that this property may be distinctive of RBR E3s. Future studies aimed at understanding the determinants within RBRs that specify the type of product generated are needed to further our understanding of RBR mechanism and function.

## Unanswered questions

The distinct features of the RBRs indicate that attempts to extrapolate from knowledge of the other better characterized classes of E3 ligases are likely to fail. This opens a host of unanswered questions regarding RBR function. Which E2s work to transfer ubiquitin to RBR E3s and what are the determinants and features of this step? To date, the cohort of E2s identified as working with RBR-type ligases most often includes the E2s UbcH7 and UbcH8 [10]. UbcH7 (and probably UbcH8 by analogy) can only transfer ubiquitin via a transthiolation reaction and are therefore specialized E2s for HECT-type transfer mechanisms [3]. Even though it is a RING domain (RING1) of RBRs that binds E2s directly, the RBR version of the domain appears to bind E2s with higher affinity than do canonical E3 RING domains: complexes of UbcH7 and RING1s can be identified by pull-down and co-immunoprecipitation experiments [28]. What are the determinants and consequences of stronger E2 binding and what features of UbcH7 and UbcH8 makes them preferred RBR-binding partners? How do RBR ligases recognize and bind substrates? Substrate binding has been reported for almost every domain from RING1 to RING2 and in regions outside the RBR domain [10]. Given the variety of substrate binding modes documented for other families of E3 ligases, it seems unlikely that RBR ligases will have a common mechanism for substrate binding. The RBR ligase Parkin has been found to associate with SCF-type ubiquitin ligases, raising the possibility that adapter proteins may provide substrate specificity for a subset of these E3s [43]. Future studies informed by the new insights regarding the distinctive mode of action for RBR E3s will no doubt shed light on these and other questions surrounding RBR function.
